# Efficacy and safety in clinical practice of a rilpivirine, tenofovir and emtricitabine single-tablet regimen in virologically suppressed HIV-positive patients on stable antiretroviral therapy

**DOI:** 10.7448/IAS.18.1.20037

**Published:** 2015-07-30

**Authors:** Nicola Gianotti, Andrea Poli, Silvia Nozza, Vincenzo Spagnuolo, Giuseppe Tambussi, Simona Bossolasco, Paola Cinque, Myriam Maillard, Massimo Cernuschi, Laura Galli, Adriano Lazzarin, Antonella Castagna

**Affiliations:** 1 Dipartimento di Malattie Infettive, San Raffaele Scientific Institute, Milan, Italy; 2 School of Infectious Diseases, Università Vita-Salute San Raffaele, Milan, Italy

**Keywords:** single-tablet regimen, rilpivirine, tenofovir, emtricitabine, efavirenz, nevirapine, protease inhibitors, simplification regimen

## Abstract

**Introduction:**

Switching to a rilpivirine, tenofovir and emtricitabine (RTE) single-tablet regimen (STR) has been evaluated in a limited number of virologically suppressed patients. The aim of this study was to describe clinical outcomes in HIV-positive patients switched from a suppressive antiretroviral regimen to RTE STR in routine clinical practice.

**Methods:**

In this retrospective study of antiretroviral-treated patients with <50 copies of HIV RNA/mL switched to RTE STR, virological failure (VF) was defined as two consecutive measurements of ≥50 copies/mL or a single measurement of ≥50 copies/mL followed by any change in treatment. Treatment failure (TF) was defined as VF or discontinuation of the STR for any reason. Univariate mixed-linear models were used to identify differences in laboratory parameters over time.

**Results and discussion:**

The analysis involved 307 patients (83% males) with a median age of 45.8 years (interquartile range (IQR 39.3–50.9), who were followed up for a median of 7.4 months (IQR 4.6–10.9). VF occurred in three patients (1%) switched from a protease inhibitor (PI)-based regimen, after a median of 2.6 months (IQR 1.6–3.0), and TF in 34 patients (11%) after a median of three months (IQR 1.4–5.8), 24 of whom (71%) were receiving a PI-based regimen at baseline. Overall, there was a slight but statistically significant improvement in the mean monthly change from baseline in CD4+ cell counts (*p*=0.027), the CD4+/CD8+ ratio (*p*=0.0001), and Hb (*p*=0.024), alanine amino transferase (ALT) (*p*=0.009), total bilirubin (*p*<0.0001), indirect bilirubin (*p*<0.0001), total cholesterol (*p*<0.0001) and triglyceride (*p*<0.0001) levels. There was also a slight but statistically significant increase in serum creatinine (*p*=0.0004), aspartate amino transferase (AST) (*p*=0.001) and liver fibrosis index (FIB-4) (*p*=0.002), and a decrease in eGFR_creat_ (*p*<0.0001) and high-density lipoprotein (HDL) cholesterol (*p*<0.0001) values. The study limitations include its retrospective design, the relatively short follow-up, and the absence of data concerning the severity of clinical adverse events; however, it does provide new information concerning the laboratory changes that occur in patients switching from PI-based or PI-sparing regimens to RTE STR.

**Conclusions:**

The study findings confirm the efficacy and safety in clinical practice of switching to RTE STR in virologically suppressed patients receiving other antiretrovirals.

## Introduction

The combination of rilpivirine, tenofovir and emtricitabine (RTE) proved to be effective safe and well tolerated in registration trials in patients starting a first-line regimen [1,2]. However, a switch from a boosted protease inhibitor (PI/r)-based regimen to a fixed dose combination (FDC) of RTE has only been evaluated in one randomised clinical trial [3], and a switch from regimens not including PIs/r to an RTE single-tablet regimen (STR) has only been studied in small non-controlled trials [4–9].

Studies of a switch to this STR from non-nucleoside reverse transcriptase inhibitor (NNRTI)-based regimens concentrated on the efficacy data because pharmacokinetic issues raise concerns in switching from first-generation NNRTIs to rilpivirine. Rilpivirine is a substrate of the CYP3A4 subunit of cytochrome p450, and efavirenz (EFV) and nevirapine (NVP) are inducers of this metabolic pathway. It has been shown that exposure to rilpivirine after switching from EFV is initially lower than that observed when rilpivirine is started without previous exposure to EFV in healthy HIV-negative adults [10]. One clinical trial found that switching from NVP did not have a clinically relevant effect on rilpivirine exposure in most patients, and that there was no need for an increased rilpivirine dose increase, additional HIV-1 RNA measurements, or therapeutic drug monitoring [7]. An open-label study in which 49 subjects were switched from an EFV-based STR to a RPV-based STR found that all remained suppressed after 12 and 24 weeks, 46 (93.9%) subjects remained suppressed after 48 weeks, and virological failure (VF) occurred in two patients (4.1%) with no emergence of resistance. EFV concentrations were above the 90th percentile of the inhibitory concentration (IC_90_) for several weeks after EFV discontinuation, and RPV exposure was in the range observed in phase III studies within approximately two weeks of the switch [4].

Switching to RTE STR has only been evaluated in a limited number of virologically suppressed patients on stable treatment. The aim of this study was to describe clinical outcomes in HIV-positive patients switched from suppressive antiretroviral regimens to RTE STR in routine clinical practice in order to provide useful information for everyday patient management.

## Methods

This retrospective study involved all of the patients with <50 copies of HIV RNA/mL receiving antiretroviral treatment at the Department of Infectious Diseases at San Raffaele Scientific Institute (Milan, Italy) who were switched to RTE STR in the context of routine clinical practice. VF was defined as two consecutive measurements of ≥50 copies/mL or a single measurement of ≥50 copies/mL followed by any change in anti-retroviral treatment. Treatment failure (TF) was defined as VF or discontinuation of the STR for any reason. Follow-up began from the start of the RTE STR (baseline) and finished at the time of its discontinuation or data freezing (23 July 2014), whichever came first. The estimated glomerular filtration rate (eGFR_creat_) was calculated using the CKD-EPI formula [11] and creatinine values, and the liver fibrosis FIB-4 index was calculated as previously described [12].

### Statistical analysis

The descriptive data are expressed as median values (and interquartile range (IQR)) or frequencies and percentages as appropriate. Changes from baseline in laboratory values were evaluated after six months and throughout the rest of the follow-up; the subjects who had been observed for less than six months after the switch were not analysed for change from baseline to month six.

The patients’ baseline characteristics were compared using Wilcoxon's rank-sum test for continuous and the chi-squared or Fisher's exact test for categorical variables. Univariate mixed-linear models were used to estimate unadjusted mean monthly changes (slopes with the corresponding standard error, ±SE) from baseline in laboratory parameters over time and identify differences between baseline PI-including versus PI-sparing regimens.

All of the statistical tests were two-sided at 5% level, and were performed using SAS software, release 9.2 (SAS Institute).

## Results and discussion

Table 1 shows the baseline characteristics of the 307 patients (83% males), aged 45.8 years (39.3–50.9) included in the analysis: 180 (59%) were receiving a PI-based regimen (48 (27%) darunavir/ritonavir, 47 (26%) atazanavir/ritonavir, 42 (23%) unboosted atazanavir, 38 (21%) lopinavir/ritonavir, and 5 (3%) fosamprenavir/ritonavir) and 127 (41%) a PI-sparing regimen (91 (72%) EFV, 14 (11%) NVP, 9 (7%) raltegravir, 4 (3%) etravirine, 1 (1%) maraviroc, and 8 (6%) a third nucleoside reverse transcriptase inhibitor (NRTI)); 227 (74%) were already receiving tenofovir (TDF).

**Table 1:** Baseline characteristics of patients switched to a single-table regimen (STR) of rilpivirine, tenofovir and emtricitabine (RTE)

	Overall (*n*=307)	On a PI-based regimen (*n*=180)	On a PI-sparing regimen (*n*=127)	*p*
Age (years)	45.8 (39.3–50.9)	45.8 (39.8–50.5)	46.1 (38.4–51.6)	0.945
Gender (male)	256 (83%)	143 (79%)	113 (89%)	**0.030**
HIV risk factor (*n*, %)				0.094
MSM	153 (50%)	87 (48%)	66 (52%)	
Heterosexual	68 (22%)	47 (26%)	21 (17%)	
IVDU	26 (8%)	17 (10%)	9 (7%)	
Other/not known	60 (20%)	29 (16%)	31 (24%)	
Years since HIV diagnosis	9.1 (4.7–15.8)	9.1 (4.3–15.5)	9.1 (5.1–16.3)	0.492
Years of ART	5.2 (2.5–12.0)	5.1 (2.5–11.3)	6.1 (2.6–13.2)	0.572
Years with undetectable viral load	3.6 (1.7–6.3)	3.2 (1.7–10.3)	4.0 (1.6–6.7)	0.222
HCV-Ab (*n* , %)				0.252
Positive	45 (15%)	31 (17%)	14 (11%)	
Negative	240 (78%)	138 (77%)	102 (80%)	
Unknown	22 (7%)	11 (6%)	11 (9%)	
HBsAg (*n* , %)				0.070
Positive	23 (7%)	18 (10%)	5 (4%)	
Negative	230 (75%)	135 (75%)	95 (75%)	
Unknown	54 (18%)	27 (15%)	27 (21%)	
Previous diagnosis of AIDS (*n* , %)	32 (10%)	19 (11%)	13 (10%)	0.999
Nadir CD4+ count (cells/µL)	290 (202–397)	272 (166–369)	322 (233–441)	**0.003**
Highest viral load before starting ART (*n* , %)				0.607
> 100,000 copies/mL	84 (27%)	52 (29%)	32 (25%)	
≤ 100,000 copies/mL	114 (37%)	68 (38%)	46 (36%)	
Unknown	109 (36%)	60 (33%)	49 (39%)	
On treatment with TDF (*n* , %)	227 (74%)	118 (66%)	109 (86%)	**<0.0001**
Reason for switching to RTE				**<0.0001**
Simplification	151 (49%)	121 (67%)	30 (24%)	
Toxicity from central nervous system	61 (20%)	1 (1%)	60 (47%)	
Dyslipidaemia	36 (12%)	26 (14%)	10 (8%)	
Other reasons	59 (19%)	32 (18%)	27 (21%)	
CD4+ count (cells/µL)	654 (516–846)	652 (514–830)	681 (519–868)	0.313
CD4 + /CD8 +	0.80 (0.59–1.09)	0.76 (0.57–1.02)	0.87 (0.61–1.18)	**0.033**
Hb (g/dL)	15.1 (14.1–15.7)	15.2 (13.9–15.7)	15.1 (14.4–15.7)	0.853
PLT (10^9^/L)	219 (186–256)	222 (180–259)	214 (190–255)	0.943
AST (UI/L)	22 (17–29)	23 (17–29)	22 (17–31)	0.838
ALT (UI/L)	30 (22–42)	29 (23–42)	31 (22–42)	0.624
ALP (UI/L)	86 (70–106)	84 (69–101)	89 (74–115)	**0.037**
FIB-4	0.86 (0.61–1.16)	0.87 (0.60–1.15)	0.86 (0.62–1.19)	0.982
Gamma GT (UI/L)	30 (20–47)	26 (18–40)	37 (25–65)	**<0.0001**
Total bilirubin (mg/dL)	0.51 (0.33–1.25)	0.99 (0.48–2.20)	0.34 (0.24–0.43)	**<0.0001**
Direct bilirubin (mg/dL)	0.17 (0.12–0.34)	0.27 (0.14–0.46)	0.13 (0.10–0.16)	**<0.0001**
Indirect bilirubin (mg/dL)	0.34 (0.21–0.88)	0.70 (0.32–1.67)	0.21 (0.13–0.29)	**<0.0001**
Creatinine (mg/dL)	0.82 (0.70–0.93)	0.83 (0.70–0.94)	0.81 (0.71–0.92)	0.763
eGFR (mL/min/1.73m^2^)	104 (94–113)	104 (95–112)	105 (93–114)	0.488
Glucose (mg/dL)	84 (78–91)	84 (77–91)	85 (81–92)	**0.043**
Total cholesterol (mg/dL)	191 (162–221)	190 (161–221)	192 (164–219)	0.806
LDL cholesterol (mg/dL)	118 (94–140)	120 (94–142)	115 (95–140)	0.956
HDL cholesterol (mg/dL)	47 (41–56)	44 (36–55)	50 (43–60)	**0.002**
Total/HDL cholesterol	4.20 (3.36–4.91)	4.18 (3.22–5.06)	4.27 (3.45–4.87)	0.779
Triglycerides (mg/dL)	115 (82–167)	126 (87–173)	106 (77–154)	0.054
Calcium (mmol/L)	2.29 (2.22–2.35)	2.30 (2.21–2.35)	2.28 (2.23–2.36)	0.616
Phosphorus (mmol/L)	1.00 (0.87–1.11)	0.98 (0.86–1.10)	1.01 (0.87–1.12)	0.450
Dip stick urinary protein (mg/dL)	5 (0–10)	5 (0–10)	5 (0–5)	0.880

PI: protease inhibitor; MSM: men who have sex with men; IDVU: intravenous drug use; ART: antiretroviral therapy; HCV-Ab: antibodies anti-hepatitis C virus antibodies; HBsAg: hepatitis B surface antigen; TDF: tenofovir; Hb: haemoglobin; PLT: platelet count; AST: aspartate amino transferase; ALT: alanine amino transferase; ALP: alkaline phosphatase; FIB-4: liver fibrosis index; gamma GT: gamma glutamyl transferase; eGFR: estimated glomerular filtration rate; LDL: low-density lipoprotein; HDL: high-density lipoprotein. Statistically significant differences are shown in bold.

The median follow-up was 7.4 months (4.6–10.9): 6.7 months (4.3–10.0) in the case of patients switched from a PI regimen, and 8.6 months (5.3–13.2) in the case of those switched from a PI-sparing regimen (*p*=0.003). VF occurred in three patients (1%) switched from a PI-based regimen after a median of 2.6 months (1.6–3.0), two of whom had a history of resistance to NRTIs and NNRTIs: previous genotype resistance tests in one of the three patients had shown the 184V, 74V and 190E mutations, and the same mutations were detected at VF; in the second, previous tests had shown the 184V, 67N, 70R and 215F mutations (no test results were available for the time of VF); the drug history of the third was not known, but no drug resistance mutation was found at the time of VF.

TF was observed in 34 patients (11%) after a median of three months (1.4–5.8), 24 of whom (71%) were receiving a PI-based regimen at baseline. The most frequent causes of discontinuation were gastrointestinal toxicity (six cases, all of dyspepsia/epigastric pain), followed by a reduction in eGFR_creat_ values (five cases) and neurological toxicity (four cases; headache in two, dizziness in one, depressed mood in one).

Tables 2 and 3 show the changes in various laboratory parameters during follow-up: overall, there was a slight but statistically significant improvement in CD4+ cell counts, the CD4+/CD8+ ratio, and Hb, alanine amino transferase (ALT), total bilirubin, indirect bilirubin, total cholesterol and triglyceride levels, and a slight but statistically significant worsening in creatinine, eGFR_creat_, high-density lipoprotein (HDL) cholesterol, aspartate amino transferase (AST) and FIB-4 values. The patients switched from a PI-based regimen showed a slight but statistically significant improvement in the CD4+/CD8+ ratio, and in Hb, ALT, total bilirubin, indirect bilirubin, direct bilirubin, total cholesterol and triglyceride levels, and a slight but statistically significant worsening in eGFR_creat_, HDL cholesterol, AST and FIB-4 values. The patients switched from a PI-sparing regimen showed a slight but statistically significant improvement in the CD4+/CD8+ ratio, and in ALP, glucose, total cholesterol and triglyceride levels, and a slight but statistically significant worsening in eGFR_creat_, direct bilirubin and HDL cholesterol values.

**Table 2:** Monthly slopes (±standard error) over the whole follow-up of laboratory values in patients switched to a single-table regimen (STR) of rilpivirine, tenofovir and emtricitabine (RTE)

	Overall (*n*=307)	*p*	On a PI-based regimen at baseline	*p*	On a PI-sparing regimen at baseline	*p*
CD4+ count (cells/µL)	4.1 (±1.8)	**0.027**	3.4 (±2.5)	0.182	5.0 (±2.6)	0.063
CD4 + /CD8 +	0.009 (±0.002)	**0.0001**	0.011 (±0.003)	**0.001**	0.007 (±0.003)	**0.033**
Hb (g/dL)	0.03 (±0.01)	**0.024**	0.04 (±0.02)	**0.020**	0.02 (±0.02)	0.392
PLT (10^9^/L)	0.3 (±0.5)	0.602	0.2 (±0.7)	0.744	0.3 (±0.8)	0.680
AST (UI/L)	0.8 (±0.2)	**0.001**	1.0 (±0.3)	**0.001**	0.5 (±0.3)	0.139
ALT (UI/L)	−1.2 (±0.5)	**0.009**	−2.0 (±0.6)	**0.001**	−0.2 (±0.7)	0.762
ALP (UI/L)	−0.7 (±0.5)	0.145	0.03 (±0.7)	0.965	−1.5 (±0.7)	**0.034**
FIB-4	0.02 (±0.01)	**0.002**	0.02 (±0.01)	**0.012**	0.02 (±0.01)	0.063
Gamma GT (UI/L)	−0.3 (±1.0)	0.749	1.3 (±1.4)	0.349	−2.3 (±1.5)	0.131
Total bilirubin (mg/dL)	−0.06 (±0.02)	**<0.0001**	−0.13 (±0.02)	**<0.0001**	0.03 (±0.02)	0.176
Direct bilirubin (mg/dL)	−0.002 (±0.003)	0.472	−0.012 (±0.004)	**0.001**	0.011 (±0.004)	**0.008**
Indirect bilirubin (mg/dL)	−0.06 (±0.01)	**<0.0001**	−0.12 (±0.02)	**<0.0001**	0.02 (±0.02)	0.339
Creatinine (mg/dL)	0.005 (±0.001)	**0.0004**	0.004 (±0.002)	**0.048**	0.005 (±0.002)	**0.003**
eGFR (mL/min/1.73m^2^)	−0.5 (±0.1)	**<0.0001**	−0.4 (±0.2)	**0.012**	−0.6 (±0.2)	**0.0003**
Glucose (mg/dL)	−0.2 (±0.1)	0.112	0.1 (±0.2)	0.639	−0.6 (±0.2)	**0.006**
Total cholesterol (mg/dL)	−2.3 (±0.4)	**<0.0001**	−2.3 (±0.5)	**<0.0001**	−2.3 (±0.6)	**<0.0001**
LDL cholesterol (mg/dL)	−0.4 (±0.4)	0.384	−0.4 (±0.6)	0.474	−0.3 (±0.6)	0.604
HDL cholesterol (mg/dL)	−0.9 (±0.1)	**<0.0001**	−0.6 (±0.2)	**0.0001**	−1.3 (±0.2)	**<0.0001**
Total/HDL cholesterol	−0.001 (±0.005)	0.921	0.008 (±0.008)	0.312	−0.006 (±0.007)	0.382
Triglycerides (mg/dL)	−5.1 (±1.0)	**<0.0001**	−6.2 (±1.4)	**<0.0001**	−3.6 (±1.5)	**0.017**
Calcium (mmol/L)	0.001 (±0.001)	0.631	0.001 (±0.002)	0.539	−0.00003 (±0.002)	0.988
Phosphorus (mmol/L)	−0.001 (±0.002)	0.580	0.002 (±0.003)	0.389	−0.0002 (±0.003)	0.933
Dip stick urinary protein (mg/dL)	0.3 (±0.2)	0.148	0.4 (±0.4)	0.238	0.3 (±0.3)	0.401

PI: protease inhibitor; Hb: haemoglobin; PLT: platelet count; AST: aspartate amino transferase; ALT: alanine amino transferase; ALP: alkaline phosphatase; FIB-4: liver fibrosis index; Gamma GT: gamma glutamyl transferase; eGFR: estimated glomerular filtration rate; LDL: low-density lipoprotein; HDL: high-density lipoprotein. Statistically significant differences are shown in bold.

**Table 3:** Median (IQR) changes from baseline to month 6 of follow-up in laboratory values of patients switched to a single-table regimen (STR) of rilpivirine, tenofovir and emtricitabine (RTE)

	Overall (*n*=238)	*p*	On a PI-based regimen at baseline (*n*=136)	*p*	On a PI-sparing regimen at baseline (*n*=102)	*p*
CD4+ count (cells/µL)	32 (−51–105)	**0.008**	21 (−51–104)	0.082	43 (−50–105)	0.053
CD4 + /CD8 +	0.06 (−0.03–0.14)	**0.0004**	0.07 (−0.02–0.15)	**0.002**	0.05 (−0.07–0.13)	0.126
AST (UI/L)	5 (−1–11)	**<0.0001**	6 (0–14)	**<0.0001**	4 (−3–9)	**0.006**
ALT (UI/L)	−5 (−19–3)	**<0.0001**	−11 (−26 to −1)	**<0.0001**	−3 (−10–5)	0.295
ALP (UI/L)	−10 (−21–2)	**<0.0001**	−6 (−18–5)	**0.027**	−13 (−23–2)	**<0.0001**
FIB-4	0.07 (−0.06–0.29)	**0.003**	0.08 (−0.06–0.30)	**0.031**	0.07 (−0.06–0.29)	0.053
Gamma GT (UI/L)	−5 (−15–3)	**<0.0001**	1 (−5–8)	0.645	−15 (−26 to −8)	**<0.0001**
Total bilirubin (mg/dL)	0.06 (−0.69–0.27)	0.198	−0.42 (−1.83–0.4)	**0.001**	0.20 (0.09–0.40)	**<0.0001**
Direct bilirubin (mg/dL)	0.03 (−0.08–0.10)	**0.006**	−0.06 (−0.28–0.08)	0.101	0.07 (0.03–0.12)	**<0.0001**
Indirect bilirubin (mg/dL)	0 (−0.56–0.18)	0.999	−0.40 (−1.39–0.04)	**<0.0001**	0.14 (0.05–0.30)	**<0.0001**
Creatinine (mg/dL)	0.05 (−0.01–0.12)	**<0.0001**	0.03 (−0.03–0.10)	**0.042**	0.07 (−0.01–0.14)	**0.0001**
eGFR (mL/min/1.73m^2^)	−4 (−11–1)	**<0.0001**	−2 (−10–2)	**0.009**	−5 (−11–0)	**<0.0001**
Glucose (mg/dL)	−2 (−10–7)	0.187	1 (−7–10)	0.826	−3 (−11–2)	**0.017**
Total cholesterol (mg/dL)	−19 (−37 to –3)	**<0.0001**	−18 (−35–0)	**<0.0001**	−23 (−39 to −9)	**<0.0001**
LDL cholesterol (mg/dL)	−5 (−22–8)	**0.033**	−4 (−21–11)	0.332	−9 (−24–5)	0.054
HDL cholesterol (mg/dL)	−7 (−12 to –2)	**<0.0001**	−3 (−10–2)	**0.007**	−8 (−15 to −4)	**<0.0001**
Total/HDL cholesterol	0 (0–0)	0.133	0 (0–0)	0.424	0 (−0.14–0)	0.286
Triglycerides (mg/dL)	−17 (−47–1)	**<0.0001**	−22 (−57–2)	**<0.0001**	−13 (−29–7)	**0.001**
Calcium (mmol/L)	0.01 (−0.09–0.09)	0.315	−0.02 (−0.09–0.05)	0.109	0.01 (−0.09–0.10)	0.892
Phosphorus (mmol/L)	0.03 (−0.11–0.14)	0.082	0.02 (−0.12–0.13)	0.328	0.03 (−0.10–0.15)	0.169
Dip stick urinary protein (mg/dL)	0 (–5–0)	0.419	0 (−5–0)	0.201	0 (−3–5)	0.999

PI: protease inhibitor; Hb: haemoglobin; PLT: platelet count; AST: aspartate amino transferase; ALT: alanine amino transferase; ALP: alkaline phosphatase; FIB-4: liver fibrosis index; Gamma GT: gamma glutamyl transferase; eGFR: estimated glomerular filtration rate; LDL: low-density lipoprotein; HDL: high-density lipoprotein. Statistically significant differences are shown in bold.

The VF and treatment discontinuation results of this study are similar to (or better than) those reported in prospective clinical trials. In the SPIRIT study, 90% of the patients switched from a PI/r-based regimen to an RTE STR maintained <50 copies/mL at week 48 at the snapshot analysis, and VFs were observed in 2.5% of patients [3]. All 32 subjects enrolled in an open-label single-centre study of HIV-1-positive adults with <50 copies of HIV-1 RNA/mL receiving TDF/emtricitabine and NVP who were willing to simplify their regimen to RTE remained virologically suppressed for 24 weeks, but three discontinued RTE for reasons other than VF: trough rilpivirine concentrations were above the mean trough concentrations observed in phase III studies by the end of the first week after the switch [5]. In a 48-week, phase IIb, open-label, multicentre study, VF (with no emergence of resistance) occurred in 2/49 subjects (4.1%) after a switch from an EFV, TDF and emtricitabine STR to an RTE STR [4]. EFV concentrations were above the 90th percentile of inhibitory concentration for several weeks after drug discontinuation, and RPV exposure was in the range observed in phase III studies approximately two weeks after the switch; none of the subjects discontinued the study due to an adverse event. Other small non-controlled trials have provided similar results [6–9].

Although we do not have any supporting pharmacokinetic data, our results seem to indicate that switching from EFV or NVP to rilpivirine is safe: the absence of VFs in this group suggests that EFV or NVP maintain higher than minimally effective concentrations (and hence significant antiviral activity) during the period before rilpivirine reaches effective steady state concentrations.

Two of the three VFs in our study occurred in patients with a history of drug resistance to NRTIs and NNRTIs: this was not unexpected and underlines that switching from a high to a low genetic barrier regimen should only be offered to patients who have never failed on NRTIs or NNRTIs. The third VF occurred in the absence of drug resistance, which suggests poor adherence to treatment.

We found that the switch to RTE was associated with an improvement in total cholesterol and triglyceride levels regardless of whether the patients were switched from a PI-based or PI-sparing regimen; the study design does not allow us to ascertain whether these changes in lipid profiles are mainly due to the withdrawal of the PI or the introduction of TDF. It must also be underlined that HDL cholesterol levels decreased and the total/HDL cholesterol ratio did not significantly change: a longer follow-up may clarify the net benefit of the switch in terms of lipid profiles.

The significant reduction in plasma bilirubin concentrations observed in the patients switched from PIs is clearly due to the withdrawal of atazanavir. However, the slight but statistically significant increase in direct bilirubin in the patients switched from a PI-sparing regimen, and the slight but statistically significant increase in FIB-4 values in those switched from a PI-based regimen deserve attention and suggest the need for a careful follow-up; the design of the study does not allow us to conclude that these changes were due to the switch, but this possibility cannot be ruled out. These findings have not been reported in previous studies, mainly because these did not specifically investigate changes in bilirubin or FIB-4.

Patients starting first-line antiretroviral therapy with NNRTI-based regimens typically experience a smaller increase in CD4+ cell counts than those starting with PI-based regimens [13]. There are no previous reports of changes in CD4+ cell counts or the CD4+/CD8+ ratio after switching from PIs or NNRTIs to RTE, but we found a slight but significant increase in both, which confirms that this strategy does not impair immune recovery.

The reduction eGFR_creat_ was expected, as it is known that rilpivirine increases serum creatinine levels by inhibiting the OCT2 tubular transporter, which reduces the tubular secretion of creatinine [14]. The reduction was slight and, although its clinical impact remains largely undefined, it has been shown that it occurs in the first few weeks of treatment and does not usually worsen thereafter [1,2].

The limitations of this study include its retrospective design and relatively short follow-up. However, it does provide new information concerning the laboratory changes (e.g. in CD4+ cell counts and FIB-4 values) that occur in patients switching from PI-based or PI-sparing regimens to RTE.

## Conclusions

The study results confirm the efficacy and safety in clinical practice of switching to RTE STR in virologically suppressed patients receiving other antiretrovirals. However, although the number of VFs was low (*n*=3), it highlights the risk of using this treatment strategy in patients with a history of resistance to NRTIs or with rilpivirine-associated resistance mutations.
